# ssRNA Virus and Host Lipid Rearrangements: Is There a Role for Lipid Droplets in SARS-CoV-2 Infection?

**DOI:** 10.3389/fmolb.2020.578964

**Published:** 2020-10-08

**Authors:** Francesca Pagliari, Maria Grazia Marafioti, Geraldine Genard, Patrizio Candeloro, Giuseppe Viglietto, Joao Seco, Luca Tirinato

**Affiliations:** ^1^Biomedical Physics in Radiation Oncology, German Cancer Research Center, Heidelberg, Germany; ^2^BioNEM Laboratory, Department of Experimental and Clinical Medicine, Magna Graecia University, Catanzaro, Italy; ^3^Department of Experimental and Clinical Medicine, Magna Graecia University, Catanzaro, Italy; ^4^Department of Physics and Astronomy, Heidelberg University, Heidelberg, Germany

**Keywords:** SARS-CoV-2, COVID-19, ssRNA virus, lipids, lipid droplets, NF-κB, TLRs, PPAR

## Abstract

Since its appearance, severe acute respiratory syndrome coronavirus 2 (SARS-CoV-2) has immediately alarmed the World Health Organization for its very high contagiousness and the complexity of patient clinical profiles. The worldwide scientific community is today gathered in a massive effort in order to develop safe vaccines and effective therapies in the shortest possible time. Every day, new pieces of SARS-CoV-2 infective puzzle are disclosed. Based on knowledge gained with other related coronaviruses and, more in general, on single-strand RNA viruses, we highlight underexplored molecular routes in which lipids and lipid droplets (LDs) might serve essential functions in viral infections. In fact, both lipid homeostasis and the pathways connected to lipids seem to be fundamental in all phases of the coronavirus infection. This review aims at describing potential roles for lipid and LDs in host–virus interactions and suggesting LDs as new and central cellular organelles to be investigated as potential targets against SARS-CoV-2 infection.

## Introduction

The rapid and global spread of a novel coronavirus (CoV) called severe acute respiratory syndrome coronavirus 2 (SARS-CoV-2), emerged in Hubei Province in China (Zhang and Holmes, 2020), has immediately alarmed the World Health Organization (WHO) for the high contagiousness and the complexity of patient clinical profiles associated with the disease, referred to as coronavirus disease 2019 (COVID-19). This has induced the WHO to declare COVID-19 a pandemic in March 2020 (World Health Organization [WHO], 2020). To date (August 2020), the total confirmed cases are more than 24 million according to data provided by the Center for Systems Science and Engineering at Johns Hopkins University^1^.

Such a situation forced almost all governments to take strong and resolute decisions in some cases culminating in the country lockdown with enormous health, social, and economic effects, the consequences of which will be visible for a long time and nowadays are perhaps difficult to quantify. This has prompted the scientific community to make a considerable effort to quickly gain knowledge about not only SARS-CoV-2–induced pathophysiology but also its biology in terms of virus–host biomolecular and biochemical interactions to identify potential targets of intervention.

Currently, SARS-CoV-2 treatments rely on knowledge gained from other human pathogenic CoV and, more in general, single-strand RNA (ssRNA) virus infections. Although specific signatures characterize each group, ssRNA viruses share similar biological mechanisms regarding their replication and the strategies to exploit and subvert host metabolic pathways, and for some of them, similar pathologies are also triggered (De Albuquerque et al., 2006; Josset et al., 2013; Menachery et al., 2014; Pombo and Sanyal, 2018). Interestingly, modulation of the host lipid metabolism represents a common feature of several viruses (Chukkapalli et al., 2012). Lipids are also involved as immune-metabolic mediators in the innate immune responses, whose functions are crucial in determining the extent of virulence and its outcomes, as they represent the first line of host defenses against invading pathogens (Frieman et al., 2008). Moreover, host lipid reprogramming can also be a host protective response to counteract viral infections.

In light of this, after a brief overview of β-CoVs, here we have reviewed most of the knowledge linking mainly positive-sense (+)ssRNA viruses, especially CoVs, to the host lipid rearrangements, with a highlight on small multifunctional organelles named lipid droplets (LDs) as potential and underexplored targets for future, but not limited to, COVID-19 studies. Moreover, the possible interactions of lipid metabolism with the innate immune responses are also discussed. We refer readers to many excellent and comprehensive reviews and papers disseminated throughout the text providing the mechanistic details of the mentioned processes. In fact, the overall goal of this review is to draw insights and offer hints to expand knowledge on aspects that might play a role in SARS-CoV-2 virulence and other potential future pandemics. This may be helpful for developing new combined approaches interfering with specific steps of the viral life cycle.

The need to discover new strategies for SARS-CoV-2 treatment is reinforced by the fact that ssRNA viruses can rapidly mutate, and even though not all mutations are biologically and pathogenically relevant, this creates concerns for the successful realization of a vaccine in a short period.

To deepen knowledge and understanding of CoV biological interactions with the host cells, including the lipid metabolic rearrangements, will allow shedding light on the mechanisms of infection at multiple levels and will help to improve the current treatment options and to design more effective and promptly available strategies, reducing the impact on public health and supporting the development of safe and effective vaccines.

## Overview of Coronaviruses

CoVs are a group of viruses belonging to the family of Coronaviridae and including four genera: α-, β-, γ-, and δ-CoVs. They share some structural and molecular features, such as the envelope, the (+)ssRNA molecule of approximately 30 kilobases, and an endoribonuclease activity (Fehr et al., 2016). CoVs can infect avian and mammalian hosts with the ability to cross the interspecies barriers and for zoonotic transmission causing respiratory, hepatic, gastrointestinal, renal, blood, and central nervous system diseases (Puelles et al., 2020). Currently, seven CoVs have been identified as being able to infect humans. In particular, three of them (all belonging to β-CoVs) gained intense attention as they were recognized as the causative agents of the outbreaks of the severe acute respiratory syndrome (SARS) that emerged in 2002 and called SARS-CoV and of Middle East respiratory syndrome (MERS) that occurred in 2012 and named MERS-CoV. More recently, in December 2019, a novel β-CoV has been identified in China and named SARS-CoV-2 because of its genetic similarity with SARS-CoV (Zhang and Holmes, 2020). Genome-phylogenetic analysis suggests that these three closely related CoVs (∼79% and 50% sequence identity between SARS-CoV-2 and SARS-CoV, and SARS-CoV-2 and MERS-CoV, respectively) (Lu et al., 2020) are most likely derived from bats with an intermediate “amplifying” host that has been potentially identified in masked palm civets and camels for SARS-CoV and MERS-CoV respectively (Zhang and Holmes, 2020) and in Malayan pangolins for SARS-CoV-2 (Xiao et al., 2020). Of note, current data on SARS-CoV-2 do not allow to exclude the possibility for a silent human-to-human transmission earlier than the evidence of the outbreak in China (Zhang and Holmes, 2020).

These highly pathogenic human CoVs induce dysfunction in host immune responses associated with respiratory diseases, which can evolve, in more severe cases, in acute respiratory distress syndrome (ARDS) and can be associated with cytokine storm and ultimately can lead to case fatality (Petrosillo et al., 2020).

The overall case fatality rate reported is ∼34% for MERS, followed by SARS ∼10%, while it is still uncertain for the ongoing pandemic of COVID-19. However, current estimates suggest that it might be lower than the other two, more male gender and age associated (Jin et al., 2020; Petrosillo et al., 2020). However, epidemiologic analyses reveal that SARS-CoV-2 is more infectious than SARS- and MERS-CoV, showing higher transmissibility among individuals (Petrosillo et al., 2020).

**FIGURE 1:**
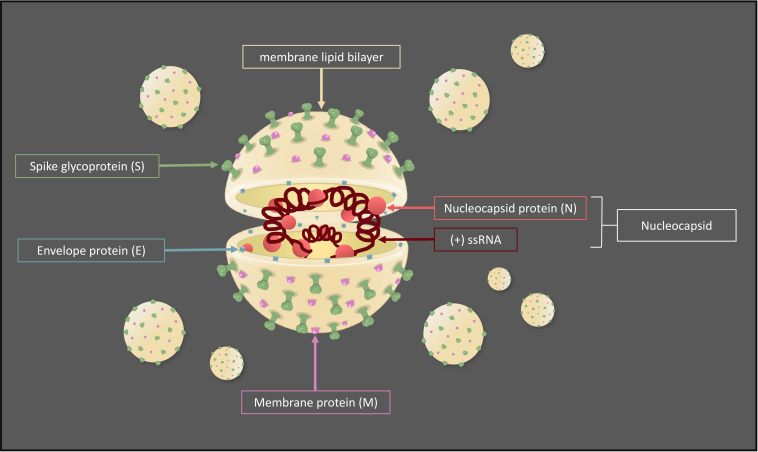
Typical coronavirus structure.

In general, CoVs are structurally composed by a nucleocapsid (N), which includes the (+)ssRNA and N proteins, surrounded by a lipid bilayer (envelope) containing envelope (E), spike (S), and membrane (M) structural proteins (NSPs), required to promote and drive the viral internalization and cytoplasmic assembly (Figure 1) (Bartlam et al., 2005). In particular, the S glycoprotein forms spike complexes protruding from the envelope and conferring a typical corona-like appearance (Wrapp et al., 2020). It shows a strong affinity to host cell receptor angiotensin-converting enzyme 2 (ACE2), highly expressed on the lung cell surface, and recently it has been demonstrated that SARS-CoV and SARS-CoV-2 bind the same host receptor. Nevertheless, the two S proteins differ in a few key amino acid residues localized in the receptor-binding domain, which might explain the difference in the ability of infection (Lu et al., 2020).

CoV RNA genome is made of a 5′ cap structure and a 3′ poly (A) tail that contains a replicase–transcriptase gene, occupying the largest portion of the genome and encoding for NSPs with multiple enzymatic functions and structural and accessories genes (Lu et al., 2020).

Once inside the host cells, CoVs redirect and exploit the host cell machinery for their proper replicative cycle. As a consequence, cellular signaling and metabolic pathways are rewired to support the infection process and to escape host immune responses.

In this context, based on previous studies on ssRNA viruses sharing several similarities (Table 1), the viral life cycle strongly depends on lipid metabolism, which can be considered a cellular hallmark of the infection-associated changes, thus suggesting a possible target for potential antiviral therapies (Heaton and Randall, 2011; Pombo and Sanyal, 2018).

**TABLE 1:** Genetic characteristics, transmission, and clinical signs of ssRNA viruses.

Name	*Genus*	Genome and virion structure	Mode of transmission	Protein receptor	Mode of entry	Clinical signs	References
**Coronaviridae**							
HCoV-229E	*Alphacoronavirus*	(+)ssRNA Enveloped	Respiratory droplets and close contact	CD13	Cell plasma membrane fusion; Caveolae-mediated endocytosis	Mild self-limiting respiratory infection and pneumonia colds	Nomura et al., 2004; Goh et al., 2012; Fehr et al., 2016
HCoV-NL63	*Alphacoronavirus*	(+)ssRNA Enveloped	Close contact	ACE2	Cell plasma membrane fusion; Clathrin-mediated endocytosis	Mild self-limiting respiratory infection and pneumonia colds	Goh et al., 2012; Fehr et al., 2016; Milewska et al., 2018
HCoV-OC43	*Betacoronavirus*	(+)ssRNA Enveloped	Respiratory droplets	*N*-acetyl-9-*O*-acetylneuraminic	Cell plasma membrane fusion; Caveolae-mediated endocytosis	Mild self-limiting respiratory infection	Owczarek et al., 2018; Fehr et al., 2016
HCoV-HKU1	*Betacoronavirus*	(+)ssRNA Enveloped	Close contact	*O*-acetylated sialic acid	Cell plasma membrane fusion; Endocytotic pathway	Mild self-limiting respiratory infection	Goh et al., 2012; Fehr et al., 2016
SARS-CoV	*Betacoronavirus*	(+)ssRNA Enveloped	Direct contact with infected individuals	ACE2	Cell plasma membrane fusion; Clathrin-mediated endocytosis; Clathrin and caveolae indipendent uptake	Severe acute respiratory syndrome	Lu et al., 2008; Wang et al., 2008; Fehr et al., 2016; World Health Organization [WHO], 2020
MERS-CoV	*Betacoronavirus*	(+)ssRNA Enveloped	Primarly from infected dromedaries and camels to people; Between people after close unprotected contact	DPP4	Cell plasma membrane fusion; Clathrin mediated endocytosis	Severe acute respiratory disease	Fehr et al., 2016; Al-Qahtani et al., 2017; Liang et al., 2018; World Health Organization [WHO], 2020
SARS-CoV2	*Betacoronavirus*	(+)ssRNA Enveloped	Airborne; Respiratory droplets and close contact	ACE2	Cell plasma membrane fusion; Endocytosis mediated pathway	Severe acute respiratory disease	Wang et al., 2008; Fehr et al., 2016; World Health Organization [WHO], 2020
**Flaviviridae**							
HCV	*Hepacivirus*	(+)ssRNA Enveloped	Bloodborne	CD81; LDLR; SR-B1; CLDN1; OCLN	Receptor-mediated endocytosis	Acute and chronic hepatopathy; Liver cancer	Andrus and East, 2010; Colpitts et al., 2020; World Health Organization [WHO], 2020
DENV	*Flavivirus*	(+)ssRNA Enveloped	Mosquito-borne	Unidentified universal cell-surface receptor	Clathrin-mediated endocytosis	Acute flu-like illness; Severe dengue	Martinez-Gutierrez et al., 2011
WNV	*Flavivirus*	(+)ssRNA Enveloped	Mosquito-borne	DC-SIGN; Mannose receptor	Clathrin-mediated endocytosis	Fatal neurological disease	Colpitts et al., 2012; Vancini et al., 2013; Fares-Gusmao et al., 2019
ZIKV	*Flavivirus*	(+) ssRNA Enveloped	Mosquito-borne	DC-SIGN; TIM-1; AXL	Clathrin-mediated endocytosis	Mild, often asymptomatic illness; Congenital malformations in infants when contracted during pregnancy	Laureti et al., 2018; Fares-Gusmao et al., 2019
**Filoviridae**							
EBOV	*Ebolavirus*	(−)ssRNA Enveloped	Blood, secretions, bodily fluids of infected people; Contaminated surfaces and materials	TIM-1; NPC1	Macropinocytosis	Hemorrhagic fever	Salata et al., 2019; World Health Organization [WHO], 2020
**Orthomyxoviridae**							
IAV	*Alphainfluenzavirus*	(−)ssRNA Enveloped	Respiratory droplets	*N*-acetylneuraminic acid (sialic acid)	Receptor mediated endocytosis	Acute respiratory infections of varying severity	Edinger et al., 2014; World Health Organization [WHO], 2020

To date, no specific antiviral treatments or vaccines have yet been developed against SARS-CoV and MERS-CoV, and also for SARS-CoV-2, this will require time. However, public health concerns are globally and tremendously increasing, calling for urgent solutions dictated by the high burden of these emerging infectious diseases.

## Cellular Lipid Modulation Induced by Virus Infection

(+)ssRNA virus infection is a complex of well-coordinated mechanisms starting with the interaction of the virus with the host cell membrane and culminating in viral particle release, for which virus relies on cellular machinery and a rearrangement of cell metabolism. Among the adaptive metabolic responses, a considerable role is given to lipid metabolism, which has been established to be involved in all the viral infection stages (Stapleford and Miller, 2010; Heaton and Randall, 2011; Pombo and Sanyal, 2018). Alterations in host lipid metabolism, mainly fatty acid (FA) metabolism, sterol biosynthesis, synthesis of specific phosphoinositides, and utilization of lipid stores, serve to provide energy and substrates for virus replication and to dampen host antiviral responses (Mackenzie et al., 2007; Pombo and Sanyal, 2018).

Evidence has shown that members of the Coronaviridae family use lipids in different steps of their cycle, although the detailed mechanisms by which they modulate lipid metabolism are not resolved yet (Wang et al., 2008; Romero-Brey and Bartenschlager, 2014).

The binding and internalization of CoVs are mainly receptor-mediated endocytic processes, in which the envelope-located proteins, such as S glycoprotein, recognize and bind specific receptors on the plasma membrane of the host cell, thus allowing also for a synergistic interaction between the viral and the host lipid bilayers (Wang et al., 2008; Lorizate and Krausslich, 2011; Burkard et al., 2014).

The fundamental lipid component of cell membranes is cholesterol, which contributes to membrane fluidity, permeability, and, in general, to the execution of its functions (Zimmerberg and Gawrisch, 2006). It is present in both the viral envelope and the host cell membrane, where it is specifically accumulated in dynamic microdomains known as lipid rafts considered entry sites for some CoVs (Li et al., 2007; Wang et al., 2008). During the first steps of infection, cholesterol role is particularly important. In fact, it has been shown that the α-CoV human coronavirus 229E (HCoV-229E) interaction with the receptor CD13 on human fibroblast caused a specific accumulation of the receptor in lipid rafts. Moreover, the use of the methyl-β-cyclodextrin (MβCD), a plasma membrane cholesterol-removing agent, significantly reduced virus infection (Nomura et al., 2004). Similar results were reported for a pseudotyped SARS-CoV, which required intact lipid rafts, where ACE2 receptors were also sequestered, in order to produce efficient viral infectivity (Lu et al., 2008). Although controversy remains regarding ACE2 association with lipid rafts (Li et al., 2007; Wang et al., 2008), these cholesterol-enriched domains are commonly recognized as essential platforms for entry promotion of SARS-CoV and more in general of (+)ssRNA viruses. Down-regulation of cholesterol synthesis has been linked to reduced susceptibility to (+)ssRNA virus–induced fusion and increased activation of antiviral immunity and, in CoV-infected cells, also type I interferon (IFN)–mediated inflammation (Mackenzie et al., 2007; York et al., 2015; Pombo and Sanyal, 2018).

For this reason, in addition to MβCD, other cholesterol-interfering drugs are now drawing the attention of the scientific community for their potential antiviral actions, such as phytosterols and β-sitosterol (Baglivo et al., 2020). Also, statins (3-hydroxy-3-methylglutaryl CoA reductase inhibitors) cholesterol-lowering molecules are used and debated as antiviral treatments (Fedson, 2006). They have been already used in patients infected with hepatitis C virus (HCV) (Andrus and East, 2010), dengue (DENV) (Martinez-Gutierrez et al., 2011), Ebola (Fedson and Rordam, 2015), and influenza viruses (Mehrbod et al., 2014). Their pleiotropic effects, which include the capability to attenuate the proinflammatory response of cytokines, such as interleukin 6 (IL-6), and to decrease neutrophil influx, as well as to improve the cardiovascular complication, make them potential and intriguing candidates for CoV treatment, particularly for the new COVID-19 outbreak treatment (Weitz-Schmidt, 2002; Fedson et al., 2020). The long-term benefits of these safe, cheap, and widely prescribed drugs, also in combination therapy, for global public health, could be valuable (Fedson et al., 2020).

However, it should also be kept in mind that a tight feedback mechanism regulates cholesterol homeostasis; thus, low cholesterol levels might foster host-compensating mechanisms of which viruses might subsequently take advantage. Indeed, in the setting of antiviral strategies, timely administration can determine the efficacy of treatments, further stressing for the need for intense research for elucidating the modality and the underlying mechanisms of infection.

Upon binding, CoVs are internalized through endosomes, followed by RNA genome release into the cytosol. Intracellular viral replication and assembly usually occur in close association with cellular structures and membrane lipids of several organelles, such as endoplasmic reticulum (ER), Golgi complex, mitochondria, LDs, and lysosomes (Stapleford and Miller, 2010; Heaton and Randall, 2011). It has been reported that the interaction of viral proteins with specific lipids mediates viral genome replication and assembly. Lipids might serve as scaffolds and provide specific signals for viral protein functions as well as localization, thus allowing for proper virus replication (Stapleford and Miller, 2010).

**FIGURE 2:**
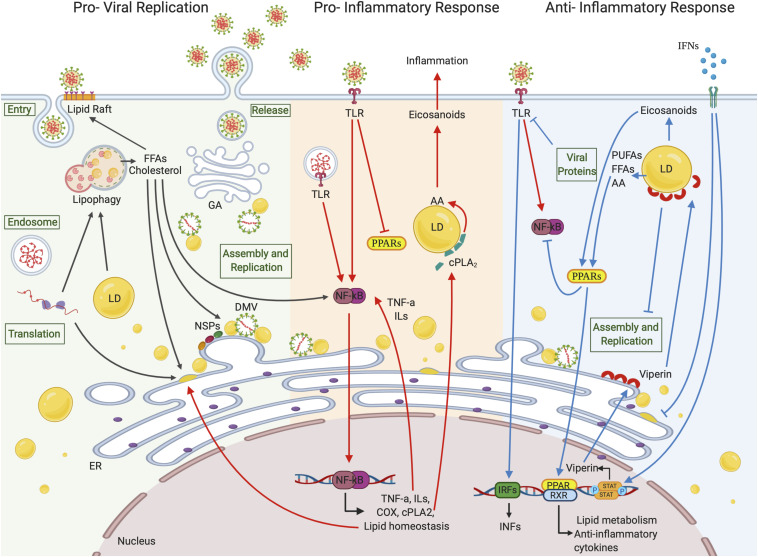
Lipid droplets involvement in (+)ssRNA virus infections. LDs seem to be involved in all the (+)ssRNA virus infection stages. During the first steps of a virus life cycle, thanks to a tight connection with ER vesicles and DMVs, LDs can serve as a platform for virus replication and assembly. LDs are also involved in the inflammation responses. Once viral signatures activate TLRs, an immune signaling cascade is initiated through the activation of the NF-κB pathway leading to the expression of downstream target genes. FFAs generated from LD metabolism rearrangement can also function as activating stimuli to NF-κB itself. cPLA2α localizes on the LD surface where it releases AA, which is converted in eicosanoids supporting inflammation. On the other side, LDs can hinder inflammation by activating PPAR anti-inflammatory signaling pathways or blocking virus replication and assembly through viperin, which localizes on LD surface. Created with BioRender.com.

These interactions, which also involve NSP subunits, lead to the extensive reorganization of host intracellular membranes and the formation of donor double-lipid membranes called double-membrane vesicles (DMVs) containing viral dsRNA (Miller and Krijnse-Locker, 2008; Romero-Brey and Bartenschlager, 2014; Lee et al., 2019) (Figure 2). DMVs in SARS-CoVs do not exist as free structures in the cytoplasm but are tightly connected to ER where they derive from, through a complex membrane network. Sometimes DMVs are referred to as “viral replication factories” because they were believed to be the sites for the synthesis of new RNA and compartments where viral components can be concentrated and protected by sensors of host innate immune surveillance (Knoops et al., 2008; Miller and Krijnse-Locker, 2008).

To sustain *de novo* membrane formation or to recycle host cell plasma membrane, (+)ssRNA viruses subvert the lipid metabolism pathways and the autophagic flux of the infected cell. To these ends, they do need to finely, and most likely temporarily, tune the processes. In fact, if, on the one hand, lipid rearrangement involves an increase in FAs and cholesterol synthesis (Heaton et al., 2010; Lorizate and Krausslich, 2011; Ketter and Randall, 2019), on the other hand, FA oxidation, cholesterol catabolism, and autophagic membrane degradation are also crucial in order to fulfill high substrate and energy demand for viral replication (Fahmy and Labonte, 2017; Pombo and Sanyal, 2018; Ketter and Randall, 2019). Recently, the lipidomic profiling of HCoV-229E–infected cells showed the up-regulation of FA synthesis and the reprogramming of linoleic acid (LA) to arachidonic acid (AA) metabolism (Yan et al., 2019). Also, infection of lung cells with MERS-CoV produced perturbations of host lipid homeostasis, with steroid biosynthesis being the most up-regulated pathway (Yuan et al., 2019). The importance of cholesterol in (+)ssRNA viral cycle is also demonstrated by the up-regulation of the key enzyme for cholesterol synthesis, 3-hydroxy-3-methylglutaryl coenzyme A reductase (HMGCR), observed in kidney cells infected with West Nile virus (WNV). Besides, in these cells, a concurrent promotion of intracellular membrane formation and inactivation of the IFN signaling pathway, most likely due to the disruption of lipid rafts after infection, occurred (Mackenzie et al., 2007). Further, it has been shown that cytosolic phospholipase A2α (cPLA2α) which releases free FAs (FFAs), such as AA [an ω-6 polyunsaturated FA (PUFA)] from membrane-associated glycerophospholipids, was critical for CoV replication, in part owing to the production of lysophospholipids required to form DMVs. In fact, cPLA2α involvement in ER-derived membrane web formation and structure has been documented (Brown et al., 2003), and its inhibition by pyrrolidine-2 negatively impacted viral RNA accumulation (Muller et al., 2018).

Taken together, these observations point out that host lipid metabolism is a key target of viral regulation in (+)ssRNA viruses and that a specific lipid profile is most likely requested to ensure productive infection. Therefore, pharmacological perturbations of this altered cellular environment may hinder infection processes by various (+)ssRNA viruses, including the novel SARS-CoV-2.

## Lipid Droplets Involvement in (+)ssRNA Virus Infection

As discussed previously, genome replication events of (+)ssRNA viruses occur in tight association with intracellular membranes on specific organelles. Among them, LDs have received particular attention, although direct studies in CoVs are still poor.

Considered only as lipid storage for a long time, their functions are increasingly being expanded. Currently, they are deemed central hubs of the lipid homeostasis, also involved in virus infection and the immune responses (Heaton and Randall, 2011; Saka and Valdivia, 2012; Zhang et al., 2017; Pereira-Dutra et al., 2019). In fact, LD formation is a frequently observed event occurring in cells from the immune system during viral infection (Saka and Valdivia, 2012; Pereira-Dutra et al., 2019).

Lipid droplets are made up of neutral lipids, including triacylglycerols (TAGs), with unsaturated and saturated chains, and cholesteryl esters (CEs), and are surrounded by a phospholipid monolayer containing several different proteins (Cermelli et al., 2006; Farese and Walther, 2009; Tirinato et al., 2017). TAGs and CEs are mainly synthesized by enzymes of glycerol phosphate and mevalonate pathways, respectively, mostly located on the membrane of ER. Among all the enzymes involved, LD generation is strongly promoted by the activity of some of them. In particular, FA synthase (FASN) yields FAs (i.e., palmitate), which are in turn elongated and converted in diacylglycerols (DAGs) and then in TAGs by diacylglycerol *o*-acyltransferase 1 (DGAT1) and 2 (DGAT2). Key enzymes involved in the cholesterol synthesis and its esters are HMGCR and acyl-CoA cholesterol acyltransferase 1 and 2 (ACAT 1 and 2). When needed, lipids are mobilized and hydrolyzed by specific lipases, such as adipose triglyceride lipase, to yield intermediary metabolites (such as DAGs, FFAs, phosphatidic acid, and sterols) necessary for the membrane synthesis, energy production through β-oxidation, and other anabolic reactions (Walther and Farese, 2012; Pol et al., 2014; Zhang et al., 2017). Cholesterol and FA depletion influences LD size and surface, which is a binding site of different proteins and enzymes, some of them involved in lipid synthesis and mobilization, such as DGAT2, cPLA2α, and other lipases (Yu et al., 1998; Kuerschner et al., 2008; Tirinato et al., 2017; Pereira-Dutra et al., 2019). Recent works have also suggested that HMGCR degradation, which generally occurs in response to high cholesterol levels, takes place on the LD surface (Hartman et al., 2010). Additionally, LDs can be used for protein storage as well as for the production of proinflammatory molecules, such as eicosanoids, deriving from the AA internalized within them (Cermelli et al., 2006; Accioly et al., 2008; Jarc and Petan, 2020).

Lipid droplets are considered to originate from ER and their composition, as well as number and size, can change depending on cell type and stimuli. Accumulation of cytoplasmic LDs in various (+)ssRNA virus–infected cells has been widely reported, and interestingly LDs have been recently proposed as platforms for virus replication and assembly (Miyanari et al., 2007; Samsa et al., 2009; Heaton and Randall, 2011) (Figure 2). In this regard, the targeting of mature capsid proteins to LDs is a common feature shared by some enveloped (+)ssRNA viruses, and it represents a critical step for RNA genome amplification (Miyanari et al., 2007; Samsa et al., 2009; Martins et al., 2019). As an example, it has been shown that capsid (core) protein of HCV localized on LD surface, together with some NSPs, and allowed replication complex-LD association, fundamental to produce infectious viral particles (Miyanari et al., 2007). Associations between clusters of membrane vesicles, DMVs, and LDs were also reported during HCV infection by Ferraris and colleagues (Ferraris et al., 2013).

Moreover, in HCV packaging, DGAT1 activity fostered the association of core protein with LDs, and treatments with DGAT1 inhibitor reduced such association and, consequently, the infectious viral particle production in hepatoma cells. DGAT1 is critical for LD biogenesis; hence, it may represent a druggable host target for antiviral interventions (Herker et al., 2010). Based on the fact that core protein–LD association is observed in different (+)ssRNA viruses, it might be interesting to evaluate and deepen if such a process also occurs in CoV- and SARS-CoV-2–infected cells, and if this is the case, this might open intriguing possibilities for developing broad-spectrum antiviral drugs.

Lipid droplet functions and their cross-talk with other cellular compartments and viral components in infectious diseases are only beginning to be investigated. Beyond being a structural scaffold for viral RNA and particle assembly, the readily available pool of lipids in LDs most likely provides host substrates needed for membrane materials and ATP production by regulating the balance between lipid accumulation (lipogenesis), storage, and mobilization (lipolysis). This regulation is influenced by viruses, and understanding the underlying mechanisms would help to control their replication cycle. For instance, HCV viral infection is able to trigger the sterol regulatory element-binding protein (SREBP) pathway, which induces *de novo* lipogenesis (Horton et al., 2002), which in turn controls the LD biogenesis, and stimulates core protein–LD formation (Li et al., 2013). Similarly, MERS-CoV caused accumulation of LDs and cholesterol, with SREBP1 and 2 being essential for viral replication. Interestingly, treatment with AM580, a retinoid derivative, inhibited SREBP-mediated lipogenesis and DMV formation (Yuan et al., 2019).

In another study, Rab18, a host protein located on LDs, was essential for DENV replication and for mediating the interaction of FASN with DENV NS3 protein, as well as FASN recruitment on LDs, in order to promote FA synthesis (Tang et al., 2014). Moreover, the inhibition of FASN by means of cerulenin or C75 (*trans*-4-carboxy-5-octyl-3-methylenebutyrolactone), which also reduced LD content, blocked DENV and HCV replication, indicating that FA production at the site of viral replication is an important requirement for viral particle production (Yang et al., 2008; Samsa et al., 2009; Heaton et al., 2010).

Although LD accumulation associated with viral replication is widely demonstrated, LD degradation is less reported, but equally considered an essential mechanism to establish a favorable microenvironment for infectious progeny virion production. Lipid mobilization from LDs serves not only to sustain viral energy requirements but also to provide viruses with specific lipids for membrane synthesis, and active lipid molecules might also be recruited in major signaling pathways activated or modified by viruses. For example, cPLA2α, which is associated with LDs and is also involved in their biogenesis, hydrolyzes membrane phospholipids, preferentially carrying AA of which LDs are rich deposits, thus releasing FFAs (Figure 2) (Yu et al., 1998; Gubern et al., 2008; Wymann and Schneiter, 2008).

Even though the hydrolytic activity of cPLA2α on LDs has not been proved yet, this suggests that impairment of CoV replication following cPLA2α inhibition, which in turn reduces DMV formation, might be mediated by LDs (Muller et al., 2018). Therefore, cPLA2α-regulated LD production may be investigated as a potential target for managing CoV infection. Moreover, free AA, generated by cPLA2α, might be essential in the membrane reorganization after CoV infection. It is known that AA further stimulates cPLA2α activity, which can favor viral replication. Further, it is also a biologically relevant FA converted into highly bioactive signaling molecules (eicosanoids) involved in inflammation with the aim of recruiting leukocytes and immune cells (Harizi et al., 2008; Ruiperez et al., 2009; Jarc and Petan, 2020). This suggests that by modulating the extent of cPLA2α activation, multiple pathways can be affected, and different effects may be obtained.

Lipid droplet catabolism can also take place through autophagy. In this process named lipophagy, LDs are delivered to the lytic compartments where lipases and hydrolyses mobilize FAs and cholesterol, which are either directed toward β-oxidation or their replenishment in LDs (Liu and Czaja, 2013) (Figure 2). Autophagic processes are interconnected with host cell defense systems, functioning as modulators of inflammation and both innate and adaptive immunity (Delgado et al., 2008; Deretic and Levine, 2009). However, several (+)ssRNA viruses, including CoVs, have evolved the ability either to escape or even exploit the autophagic machinery to promote their survival and sustain their expansion (Prentice et al., 2004; Cottam et al., 2011; Zhang et al., 2017). In a recent work, MERS-CoV infection reduced autophagy, most likely by suppressing the formation of autolysosomes (Gassen et al., 2019). Instead, lipophagy correlated with a reduction in LD size and an increase in β-oxidation in DENV-infected hepatoma cells. This resulted in efficient DENV replication, a process that was in turn affected by autophagy inhibition (Heaton and Randall, 2010).

The dual-effect (proviral or antiviral) of autophagic flux demonstrates the extreme complexity of the regulative mechanisms in the interaction virus–host cell.

To date, the role of LD biogenesis and lipophagy in β-CoV infection is largely underexplored. Whether and how the new SARS-CoV-2 benefits or is disadvantaged from these mechanisms may represent a new research field that would merit to be explored in order to add knowledge and potentially open new possibilities of intervention.

## The Innate Immune Response: Toll-Like Receptors and Links With Lipids and Lipid Droplets

As a first line of defense, infected cell respond to viral entry by activating the innate immunity, which represents a fast, although not specific, response mechanism (Akira et al., 2006). Accumulating evidence shows a strong interaction between innate immune signaling pathways and lipid metabolism regulation (Wu et al., 2016; Pombo and Sanyal, 2018; Zhang et al., 2018). Nevertheless, the interdependence of innate immune responses with lipid metabolism in general, and LD specifically, has been little investigated in CoVs.

An important role in influencing the development of the innate antiviral immunity is played by the Toll-like receptors (TLRs). TLRs are a class of proteins (collectively known as pattern recognition receptors) expressed on the leukocyte cell surface, such as macrophages, T and B cells, as well as on non-immune cells and endosomes. TLRs are able to recognize pathogen signatures (referred to as pathogen-associated molecular patterns such as viral nucleic acids and envelope proteins) and initiate the innate immune signaling cascade in order to hinder viral replication and to promote the adaptive immunity (Akira et al., 2006; Kawai and Akira, 2010). Activated downstream pathways of TLRs involve, among others, the activation of nuclear factor-κ light chain-enhancer of activated B cells (NF-κB) signaling, which culminates in the modulation both of key proinflammatory cytokines, such as tumor necrosis factor (TNF-α), ILs, and IFNs (this latter also regulated through interferon regulatory factors), and of lipid homeostasis (Alexopoulou et al., 2001; Akira and Takeda, 2004) (Figure 2). In fact, TAG synthesis and CE accumulation upon TLR activation have been reported, with FASN, DGAT1 and DGAT2, and ACAT1 and ACAT2 being the enzymes more expressed (Pereira-Dutra et al., 2019). Further, distinct membrane lipids can influence TLR signaling, and activated TLRs can be recruited into lipid rafts, highlighting a critical role of these lipid microdomains in participating in cellular immune responses (Bonham et al., 2014; Ruysschaert and Lonez, 2015; Koberlin et al., 2016).

Evidence shows that cytokines can have an indirect antiviral activity by modulating FA and cholesterol metabolism (Zhang et al., 2018). For example, type I IFNs participate in lipid metabolism regulation, on the one hand, by down-regulating cholesterol biosynthetic pathway and inducing FA oxidation to limit the availability of substrates for viral replication, reinforce IFN responses, and establish a hostile microenvironment. On the other hand, type I IFNs can also promote cholesterol import, for example, to stimulate foam cell production (York et al., 2015; Wu et al., 2016; Pombo and Sanyal, 2018). These observations emphasize the tight link between the IFN system and lipid regulation as a possible mechanism that contributes to the metabolic changes occurring upon pathogen invasion (Figure 2).

Moreover, autophagic processes, which remove viral components, can also stimulate antiviral type I IFN-mediated responses by activating endosomal TLRs, and in turn, TLRs and cytokines are autophagy modulators (Delgado et al., 2008; Deretic and Levine, 2009; Li et al., 2012).

Conversely, (+)ssRNA viruses have developed multiple strategies to subvert the host innate immune responses at various levels, including (i) the suppression or the induction of TLR activation and its signaling, (ii) the inhibition of IFN system, and (iii) the damping of autophagy-dependent activation of immunity (Munoz-Jordan et al., 2003; Li et al., 2005; Abe et al., 2007; Deretic and Levine, 2009; Al-Qahtani et al., 2017). For instance, to favor their replication cycle while eluding the immune system, SARS-CoV can encode proteins that inhibit TLR signaling. In fact, although exogenous treatment with type I IFNs can reduce SARS-CoV and HCoV-229E infections, viral strategies, mediated by viral NSP3 macrodomain, or the open reading frame (ORF) 3b, ORF 6, and N proteins, are also activated to suppress the effect of IFNs and promote rapid spreading (Kopecky-Bromberg et al., 2007; Kuri et al., 2011; Fehr et al., 2016). Also, it has been reported that, in MERS-CoV–infected macrophages, a crucial role in immunosuppression was played by the S glycoprotein, which blocked TNF-α and IL-6 production, while down-regulating TLR signaling (Al-Qahtani et al., 2017).

However, it has been shown that SARS-CoV could also stimulate TLR3 and TRL4 expression, which resulted in protective effects in a mouse model (Totura et al., 2015). Also, protection was observed in the early phase of infection, thanks to the production of type I IFN by plasmacytoid dendritic cells (pDCs), via TRL activation, in SARS-infected donor cells (Cervantes-Barragan et al., 2007). Conversely, a delay in type I IFN signaling correlated with high levels of proinflammatory cytokines and chemokines and lethality in SARS-CoV–infected mice (Channappanavar et al., 2016). Of note, conflicting reports from both SARS and COVID-19 patients, as well as from various cell culture systems productively infected, show differences in IFN and cytokine induction, which may reflect differences in patient cohorts/cell lines, as well as in temporal analysis and assay sensitivity (Frieman et al., 2008; Guo et al., 2020). While a low type I IFN activation can benefit to viral replication, an overactivation of this pathway can be harmful for the infected patients. Actually, dysregulated IFN-mediated immune responses and aberrant cytokine release are phenomena frequently observed, although not fully characterized yet, in severe SARS patients (Peiris et al., 2004; Wang et al., 2007) and, more recently, in severe COVID-19 patients usually correlated with poor prognosis (Huang et al., 2020).

Overall, these observations indicate that properly regulated TLR activation and IFN responses represent a critical aspect of the CoV-related immunopathogenesis.

In this regard, although LDs are supposed to be a platform for viral replication, they might also contribute in antiviral signaling events, suggesting a double role for these organelles, which makes the scenario more complex and less trivial than anticipated (Monson et al., 2018). In fact, LD biogenesis is a well-established event occurring in cells of the immune system following pathogen infections and inflammation. (+)ssRNA viruses are able to trigger LD accumulation and enlargement in various leukocytes as a marker of cell activation (Bozza et al., 2009; Syed et al., 2010). Further, it has been shown that, in HCV-infected human hepatoma cell lines, LDs were the colocalization site of viperin (also known as RSAD2) and HCV NSP 5A, an event resulting in limited HCV replication (Helbig et al., 2011). In fact, viperin is a protein coded by one of the interferon-stimulated genes (ISGs) either after binding of IFNs to their receptors or via an IFN-independent pathway (Seo et al., 2011). Its antiviral properties have been documented, as well as its accumulation in LDs (Hinson and Cresswell, 2009; Helbig et al., 2011; Seo et al., 2011). Saitoh et al. (2011) have shown that viperin localized to LDs and promoted the activation of the TLR 7 and 9 pathways in pDCs, and they have proposed LDs as transit points for the consequent type I IFN production. In another study, Monson and coworkers (Monson et al., 2018) highlighted how low LD content in human cell lines challenged with dsRNA viral mimics reduced IFN production, viperin expression, thus delaying the host immune response.

As reported in the previous paragraph, cPLA2α releases AA from LDs. Of note, cPLA2α is activated by several factors, including TLRs (Pindado et al., 2007; Ruiperez et al., 2009). Once produced, AA is the substrate for the synthesis of eicosanoids involved in the initiation of inflammatory cascades and in the recruitment and infiltration of neutrophils (Harizi et al., 2008; Jarc and Petan, 2020). It was shown that AA metabolic pathway was up-regulated in HCoV-229E–infected cells and that treatment with exogenous AA inhibited both HCoV-229E and MERS-CoV replication (Yan et al., 2019). This suggests another potential link between LDs in CoVs and immune responses.

Although the connection between LDs, viral infection, and the immune response has been underexploited and is only starting to attract interest from the scientific community, current knowledge supports the idea that, through TLR activation, LDs may also act as mediators participating in the induction of innate immune responses upon CoV infection. The regulatory processes involved in a proviral or antiviral role for LDs also remain to be explored.

## Linking NF-κB–Mediated Responses With Lipid Metabolism in (+)ssRNA Virus Infections

The signaling cascade initiated by TLRs leads to the activation of NF-κB pathway, which usually represents a common feature of many viral infections (Akira and Takeda, 2004; Kawai and Akira, 2006). NF-κB is a ubiquitous transcriptional factor that regulates the expression of hundreds of target genes, many of which participate in immune and inflammatory processes. NF-κB family includes five members of inducible transcription factors: NF-κB1 (also named P50), NF-κB2 (or P52), RelA (or P65), RelB, and c-Rel (Akira et al., 2006; Liu et al., 2017). Their association with inhibitory proteins, belonging to the inhibitor of κB (IκB) protein family, sequesters NF-κB factors in the cytoplasmic compartment, making them inactive. Several stimuli can induce NF-κB activation, such as exposure to cytokines (ILs and TNF) and viral infections. This results in the degradation of IκB members, followed by NF-κB dimer release and translocation to the nucleus, where it binds to DNA-specific sequences of proinflammatory genes, including a subset of ISGs (Barnes and Karin, 1997; Akira and Takeda, 2004; Pfeffer, 2011) (Figure 2). Interestingly, it has been demonstrated that viperin, which is a ISG, is involved in type I IFN production through an NF-κB–orchestrated mechanism in carcinoma cells (Moschonas et al., 2012). It may be of relevance to evaluate whether such a mechanism also occurs upon SARS-CoV-2 infection and whether LDs are entailed to some extent in this axis.

Of note, IFNs and FFAs, derived from lipid rearrangements, also lead to NF-κB signaling activation by favoring the dissociation of IκB-α, thereby initiating the proinflammatory responses (Yang et al., 2000; Baker et al., 2011). To this regard, possible links between NF-κB and lipid metabolism have been described in different cell types. For instance, it has been reported that NF-κB activation induced SREBP-1a expression in macrophages, as the SREBP1 promoter region contains an NF-κB response element, and subsequently stimulated lipogenesis and IL-1β production (Im et al., 2011). Similarly, in liver cells, exogenous FAs induced NF-κB pathway and SREBP-1c expression with LD formation (Jung et al., 2013). These observations further reinforce the connection between the innate immune responses and the lipid metabolism and LDs also through NF-κB–dependent mechanisms.

Many inflammatory lung diseases, such as asthma, are characterized by a constitutive NF-κB activation, which leads to the release of cytokines and adhesion molecules. This effect helps to recruit activated immune and inflammatory cells to the site of interest, thus amplifying and, in some cases, even exacerbating the inflammatory state (Barnes and Karin, 1997; Schuliga, 2015). Similarly, viral infections can trigger NF-κB–mediated immune and proinflammatory responses as a host defense mechanism and, at the same time, viruses can develop counter-strategies to actively modulate these networks for their purposes. In fact, prolonged NF-κB activation has been shown to be fundamental for the inflammatory immunopathology induced by β-CoVs (Smits et al., 2010; DeDiego et al., 2014). In particular, *in vitro* studies showed that SARS-CoV S protein triggered NF-κB pathway causing a marked up-regulation of it in macrophages compared to control with a simultaneous massive release of IL-6 and TNF-α (Wang et al., 2007; Dosch et al., 2009). Similar effects were induced by SARS-CoV E protein in *in vitro* and *in vivo* studies (DeDiego et al., 2014). Remarkably, these two cytokines can, in turn, further stimulate NF-κB through a positive feedback loop and increase cytokine levels, thus amplifying the responses and leading to aberrant inflammation (Barnes and Karin, 1997). Additionally, secreted ILs and TNF-α exhibit an intense regulatory action on lipid homeostasis. In fact, TNF-α is able to promote both *de novo* lipogenesis and to induce lipolysis, this latter effect also induced by IL-6, as well as to inhibit the activity of different lipid-related enzymes and to control cholesterol metabolism (Chen et al., 2009; Lehrskov and Christensen, 2019). However, other studies have shown that SARS-CoV N protein was able to either dramatically inhibit NF-κB, which in part could explain the observed inhibition of IFN synthesis or activate it. These findings suggest that NF-κB induction and its signaling might be cell context-specific and may influence distinct aspects of immune function (Kopecky-Bromberg et al., 2007; Zhang et al., 2007). Nevertheless, although accumulating data highlight a central of NF-κB as modulator of antiviral responses in β-CoVs, its involvement in lipid metabolism has not yet been fully investigated in the context of CoV infections.

As the central driver of the inflammation and innate immunity triggered by viruses, NF-κB is considered as a potential therapeutic target, and hundreds of inhibitors have been studied (Karin et al., 2004; Miller et al., 2010). Among them, it has been reported that statins reduce the levels of proinflammatory cytokines by inhibiting NF-κB activity (Hilgendorff et al., 2003). Moreover, treatments with NF-κB inhibitors, caffeic acid phenethyl ester (CAPE) and parthenolide, reduced inflammation and lung disease and increased survival after SARS-CoV infection in *in vitro* and *in vivo* studies (DeDiego et al., 2014). Another therapeutic approach explored against an α-CoV was the combination of specific NF-κB signaling inhibitors together with small molecules, called tylophorine, which resulted in interfering with the virus replication and reducing proinflammatory responses (Yang et al., 2017). However, it is worth to note that NF-κB pathway is involved in multiple cellular events and is physiologically required for the maintenance of normal immune response and cell survival; therefore, before using NF-κB–modulating drugs as a potential strategy for COVID-19 patients, it is fundamental to carefully evaluate the balance between efficacy and safety. This aspect requires intense investigations of the influenced pathways. In this respect, future integrated data from genomic, proteomic, and lipidomic analyses will help to decipher the overall functional effects of NF-κB inhibition *in vitro* and *in vivo* and to design more specific combined strategies.

## Peroxisome Proliferators Activated Receptors and Lipid Metabolism in Viral Infection

Immune and inflammation reactions are also modulated by lipid molecules through another class of receptors, called peroxisome proliferator-activated receptors (PPARs), which interfere with NF-κB signaling. PPARs represent a family of nuclear hormone transcriptional factors activated by lipid ligands, such as oxidized FAs, eicosanoids, and lysophosphatidic acid, with a significant role exerted by PUFAs (Harmon et al., 2011; Varga et al., 2011). After heterodimerization with the retinoid X receptor, they recognize and bind the peroxisome proliferator–responsive elements (PPREs) present on the promoter of target genes, thus activating them (Harmon et al., 2011; Poulsen et al., 2012).

By now, three isoforms of this family have been discovered, each of them modulating lipid homeostasis in distinct ways: PPARα, PPARβ/δ, and PPARγ (Harmon et al., 2011; Varga et al., 2011). PPARα activates FA oxidation and is highly expressed in the liver, heart, and brown adipose tissue. PPARβ/δ is mainly involved in FA catabolism in several tissues but especially in skeletal muscle, whereas PPARγ regulates adipogenesis, lipid metabolism, and storage in all tissues, mostly adipose tissue (Di Paola and Cuzzocrea, 2007; Poulsen et al., 2012).

Considered as lipid sensors able to sense cellular lipid milieu, PPARs positively regulate the expression of genes whose products are involved at different stages of FA and cholesterol metabolism, such as FA transporter proteins and acyl-CoA synthetase, which catalyzes the FA conversion in ester compounds (acyl-CoAs) (Gervois et al., 2000). As such, activated FAs are substrates for *de novo* lipid synthesis, β-oxidation, AA production, and membrane remodeling (Grevengoed et al., 2014).

Moreover, PPARs also participate in LD biogenesis (de la Rosa Rodriguez and Kersten, 2017; Souza-Moreira et al., 2019). In particular, PPARγ is proven to up-regulate the expression of genes involved in TAG synthesis and LD-associated proteins, such as perilipin (PLIN) family members. In fact, the presence of PPRE sequences in the promoter region of some PLINs has been demonstrated (Nagai et al., 2004; de la Rosa Rodriguez and Kersten, 2017; Pereira-Dutra et al., 2019).

Peroxisome proliferator-activated receptors activity is positively modulated by intracellular concentrations of ligands, and in the absence of the ligands, the association of the heterodimers with corepressors suppresses target gene transcription. This highlights the ability of these nuclear receptors to activate positive or negative regulatory responses depending on the cellular context (Ricote and Glass, 2007; Glass and Saijo, 2010).

Peroxisome proliferator-activated receptors role upon (+)ssRNA viral infection is poorly investigated, and information is mainly obtained from studies with HCV-infected cells. In general, altered production of lipid ligands induced by viral replication may influence nuclear receptor expression and activity. Nevertheless, PPAR activation and modulation of the lipid microenvironment may interfere with viral responses. In this context, an impaired expression of PPARα, most likely influenced by HCV core protein, has been reported during the infection in patient-derived liver cells (Dharancy et al., 2005). However, further studies showed that, in chronic infection of various liver cells, treatment with a PPARα antagonist, which also down-regulates hydroxymethylglutaryl-CoA (HMG-CoA) synthase gene, was able to reduce HCV replication and resulted in increased lipid and LD accumulation likely, at least in part, owing to the role exhibited by PPARα in lipid catabolism (Rakic et al., 2006). Inhibition of the PPAR signaling pathway was also observed in DENG, and WNV-infected asymptomatic donors (Fares-Gusmao et al., 2019). By contrast, a recent study from Al-Qahtani et al. (2017) reported the induction of PPARγ in MERS-infected cells, which occurred after 24 h and not at earlier stages of stimulation. PPARγ activation was mediated by S glycoprotein with concurrent inhibition of macrophage responses and suppression of proinflammatory cytokines. In this regard, PPARs can also be stimulated by AA-derived metabolites (prostaglandins and leukotrienes), that, as previously reported, are mediators of the inflammatory responses (Devchand et al., 1996; Ricote et al., 1998; Lee et al., 2003; Varga et al., 2011). Accumulating data support the interconnection of PPAR signaling with inflammation and immunity, as already well-established in the pathogenesis of some diseases, also in the context of viral infection. In fact, PPARs are expressed in vascular and immune cells, such as in dendritic cells (DCs), macrophages, and lymphocytes (Szatmari et al., 2007; Glass and Saijo, 2010; Varga et al., 2011).

Notably, PPARγ appears to be a critical regulator involved in the immune responses, and more particularly in activated macrophages, where its expression is markedly up-regulated. PPARγ up-regulation and its ability to antagonize the activities of the transcription factors, such as NF-κB (via the up-regulation of IκBα), result in the repression of genes encoding for proinflammatory cytokines, such as TNFα, IL-6, IL-8, and IL-1β, and in the induction of anti-inflammatory cytokines, such as IL-10 and transforming growth factor (Ricote et al., 1998; Croasdell et al., 2015) (Figure 2). In addition, PPAR activation can inhibit IFN production, and concurrently cytokines can modulate PPAR expression in immune cells (Ricote and Glass, 2007).

Because of the inhibition mechanism exerted by PPARγ on NF-κB signaling, it is reasonable to argue that an interplay between PPARs and TLRs exists. Indeed, although the underlying mechanisms remain elusive, it has been proposed that TLRs/NF-κB signaling may block PPARγ activation in various cell types (Dana et al., 2019). Additionally, *in vitro* and *in vivo* studies showed that upon influenza virus infection, the expression of PPARγ in alveolar macrophages was down-regulated via type I IFNs, a strategy that most likely helps to respond to viral entry quickly. However, PPARγ complete loss produced increased proinflammatory cytokine release, which led to the worsening of the pathology (Huang et al., 2019). In fact, it is believed that activated PPARs counteract the amplification of the TLR/NF-κB pathway. In this regard, it has been shown that the activation of DCs, stimulated by synthetic analogs of dsRNA, which binds TLR3, could be inhibited by PPARγ agonists and resulted in the down-regulation of NF-κB pathway (Appel et al., 2005).

These observations, although far to be conclusive, support the idea that PPARs might participate in attenuating the inflammatory and immune responses after their induction following viral infection. This event requires to be temporarily and finely regulated in an attempt to limit the excessive production of inflammatory mediators, while not wholly abolishing them to allow an adequate antiviral reaction and to mount the adaptive immunity (Bassaganya-Riera et al., 2010). Interestingly, Szatmari et al. (2007) reported that PPARγ activation in DCs resulted in the up-regulation of lipid metabolism genes and reduction in LD content in the early stages. In contrast, genes related to immune responses were modulated at later time points, thus showing a temporal link between lipid and immune regulation (Szatmari et al., 2007).

Furthermore, based on the role of cPLA2α in AA metabolism, it was shown that PPARγ activity in airway epithelial cells could be stimulated by the overexpression of cPLA2α, which consequently increased PPARγ-mediated gene transcription through induction of PPRE reporter activity, in particular IL-8 and cyclooxygenase-2 (COX-2) expression (Pawliczak et al., 2002, 2004). Indeed, evidence shows that PPARs participate in the modulation of inflammation occurring during lung inflammation and ARDS (Di Paola and Cuzzocrea, 2007; Bassaganya-Riera et al., 2010), and activated PPARs can reduce the fibrotic responses in models of lung injury (Lakatos et al., 2007), which can also occur in severe CoV and, more specifically, COVID-19 patients (Hui et al., 2005; Spagnolo et al., 2020). In this regard, PPARγ agonists have been suggested as possible antiviral therapy for pulmonary virus-induced diseases (Bassaganya-Riera et al., 2010).

Lastly, evidence shows that a gender-related expression of PPARs in cells of the immune systems exists. In fact, while androgen hormones can increase PPARα gene expression, estrogens influence PPARγ gene expression. Consequently, PPARα is usually overexpressed in male T cells, and PPARγ is more abundant in female T cells, which also exhibit a differential cytokine production. Such a hormone-specific influence on PPAR expression might influence the gender-related infection susceptibility and disease outcomes (Zhang et al., 2012; Park and Choi, 2017).

In light of these considerations, it would be appealing to elucidate the PPAR role in lipid and LD homeostasis, as well as weather and how PPARs-TLRs/NF-kB axis might regulate them, during CoV infection especially in acute phases.

## Concluding Remarks

The COVID-19 sanitary emergency has shown us that it became imperative and urgent to explore and evaluate new possible biological routes that can result in rapid and effective antiviral therapies to treat SARS-CoV-2 infection. To this purpose, the integration of clinical with molecular data and the creation of shared databases are crucial and useful to better deal with the current and the future outbreaks. This requires intense and robust research that allows unveiling the specific mechanisms of infection and identifying precise and potentially combined points of intervention.

Based on a growing body of knowledge, deregulated host lipid metabolism is a typical signature observed in many (+)ssRNA virus infections, CoVs included, as a proviral or antiviral mechanism, also having profound effects on innate immune responses. In this scenario, LDs seem to be active players at the crossroad of different ways by providing support as well as key metabolites, according to the renewed view as multifunction organelles also involved in viral replication. This evidence drove us to wonder whether a putative role for lipids and LDs in SARS-CoV-2 can also exist, and if this is the case, they might be direct or indirect druggable targets.

While waiting for safe vaccines, host-directed antiviral therapies seem to be a more promising approach against infectious diseases than pathogen-directed strategies, due to the possibility to target multiple aspects at the same time, reduce the impact of drug resistance, and develop broad-spectrum antiviral drugs able to treat different, but still similar, virus strains. Of course, this option imposes the need for a careful evaluation of the benefits, while minimizing the adverse effects for the host. Currently, several safe and cheap drugs interfering with lipids and LDs, which are host factors requested for the viral life cycle, exist and are already on the market. These could be considered and promptly tested in order to evaluate their effectiveness in SARS-CoV infections.

To date, multiple approaches have been tested on patients based on previous and limited studies, also in absence of clear data on the potential effects. This has been dictated, and intrinsically approved, by the need to rapidly manage and respond in the initial phases of this pandemic. However, now it is important both to get data from large randomized trials and to stimulate research in order to define safe therapeutic strategies only after accurate and validated studies.

## Author Contributions

LT conceived the idea. FP, MM, and LT studied the topic and wrote the first draft. GG and PC organized the literature database and contributed to draw the table and figures. GV and JS provided the ideas contributing to conception and design of the manuscript. FP and LT supervised the project. All the authors contributed to manuscript revision, read, and approved the submitted version.

## Conflict of Interest

The authors declare that the research was conducted in the absence of any commercial or financial relationships that could be construed as a potential conflict of interest.

## Footnotes

1 https://coronavirus.jhu.edu/map.html
